# Baseline Hepatitis B Immunity and Vaccination Booster Response Among Medical Residents: A Longitudinal Study in a Spanish Tertiary Hospital

**DOI:** 10.3390/vaccines14030280

**Published:** 2026-03-23

**Authors:** Victoria Salguero-Cano, Silvia Martínez-Martínez, Manuel González-Alcaide, Carmen Valero-Ubierna, Virginia Martínez-Ruiz, Mario Rivera-Izquierdo, Inmaculada Guerrero-Fernández de Alba

**Affiliations:** 1 Service of Preventive Medicine and Public Health, Hospital Universitario San Cecilio, 18016 Granada, Spain; vsalguero@correo.ugr.es (V.S.-C.); mariac.valero.sspa@juntadeandalucia.es (C.V.-U.); inmaculada.guerrero.f.sspa@juntadeandalucia.es (I.G.-F.d.A.); 2 Doctorate Program in Clinical Medicine and Public Health, University of Granada, 18016 Granada, Spain; 3 School of Medicine, University of Granada, 18016 Granada, Spain; silviamx2@correo.ugr.es (S.M.-M.); mangonalc@correo.ugr.es (M.G.-A.); 4 Department of Preventive Medicine and Public Health, University of Granada, 18016 Granada, Spain; virmruiz@ugr.es; 5 Instituto de Investigación Biosanitaria ibs.GRANADA, 18009 Granada, Spain; 6 CIBER de Epidemiología y Salud Pública (CIBERESP), 28029 Madrid, Spain

**Keywords:** hepatitis B, medical residents, booster vaccination

## Abstract

**Background:** Despite universal infant hepatitis B virus (HBV) vaccination, declining circulating anti-HBs levels are increasingly observed in young healthcare professionals, a high-risk group for occupational exposure. Although several studies have evaluated HBV antibody persistence in healthcare workers, data specifically addressing newly incorporated medical residents in the Spanish context remain limited. This study evaluated baseline anti-HBs levels and serological response to a vaccination booster dose in medical residents at a Spanish tertiary hospital. **Methods:** A retrospective longitudinal observational study was conducted among medical residents attending the Preventive Medicine Service of Hospital Universitario San Cecilio (Granada, Spain) between 2021 and 2024. Anti-HBs antibody titers were obtained at baseline and ≥10 mIU/mL were considered the conventional protective threshold. Residents with anti-HBs < 10 mIU/mL received an Engerix-B booster followed by repeat serology. Demographic and occupational variables were analyzed. Measles serostatus was collected for comparisons. **Results:** A total of 275 residents were included (mean age 25.4 years, SD = 2.3 years; 64% females). Baseline serology showed anti-HBs levels < 10 mIU/mL in 53.1% of participants. Lower baseline anti-HBs levels were associated with younger age (adjusted OR = 0.75; 95% CI: 0.64–0.88) and earlier residency year (R1–R2) (adjusted OR = 0.28; 95% CI: 0.13–0.61). Among 116 residents receiving a booster, 94.8% achieved anti-HBs ≥ 10 mIU/mL after booster administration. Measles serology was negative in 54.6% of participants. **Conclusions:** More than half of newly incorporated medical residents had anti-HBs levels below the conventional protective threshold (10 mIU/mL), yet almost all demonstrated a strong anamnestic response, supporting the persistence of immunological memory despite reduced circulating antibody concentrations. Systematic baseline screening combined with targeted booster vaccination appears to be an effective strategy to ensure occupational protection. Further research incorporating cellular immunity markers may refine future vaccination policies and booster strategies.

## 1. Introduction

Hepatitis B virus (HBV) infection remains a major global public health concern. The World Health Organization (WHO) estimated that in 2022 approximately 254 million people were living with chronic HBV infection, with about 1.2 million new infections occurring annually [1]. HBV-related mortality is estimated at around 1.1 million deaths per year, primarily due to cirrhosis and hepatocellular carcinoma [1]. Nevertheless, hepatitis B is a vaccine-preventable disease, and the widespread implementation of safe and effective vaccines has led to a substantial reduction in infection incidence in many countries [1,2,3].

Over the past three decades, hepatitis B vaccination has become one of the most successful global public health interventions. Universal infant vaccination programs have led to dramatic reductions in HBV incidence, particularly in countries where vaccination coverage exceeds 90%. In many settings, the prevalence of chronic HBV infection among younger cohorts has declined substantially compared with pre-vaccination generations. Nevertheless, the long-term persistence of vaccine-induced immunity remains an area of ongoing research, particularly among individuals vaccinated during infancy who reach adulthood several decades after primary immunization. As these vaccinated cohorts progressively enter healthcare professions, questions have emerged regarding the durability of protection and the interpretation of declining antibody titers over time.

Healthcare workers represent a significant risk group for HBV acquisition due to occupational exposure to blood and other biological fluids [1]. Percutaneous injuries, contact with contaminated sharps, and exposure to infected biological fluids represent well-documented transmission routes in healthcare environments. Although the implementation of standard precautions and vaccination policies has significantly reduced occupational HBV transmission, newly incorporated healthcare workers may still face exposure risks during the early stages of clinical training. Medical residents are particularly relevant in this context because they frequently perform invasive procedures while simultaneously undergoing occupational health screening at the start of their professional careers.

In Spain, hepatitis B vaccination is not legally mandatory for the general population; however, it is included in the national immunization schedule and systematically administered during infancy (10 μg, hexavalent DTaP-HepB-IPV-Hib vaccine). For healthcare workers and medical trainees, vaccination is strongly recommended and routinely verified upon entry into healthcare institutions as part of occupational risk prevention policies. Compliance with vaccination recommendations is generally required to ensure safe clinical practice. In Spain, hepatitis B vaccination was initially introduced for high-risk groups in the 1980s and adolescent immunization programs began regionally in the early 1990s; universal infant vaccination was progressively implemented across autonomous regions between 1992 and 1996 and was fully included in the national immunization schedule by the mid-1990s [4]. As a result, most young healthcare professionals, including medical residents (MIR, the Spanish national medical residency training program), have been vaccinated during childhood. Vaccine protection is usually assessed by determining antibodies against the HBV surface antigen (anti-HBs), with titers ≥ 10 mIU/mL considered protective, while levels below this threshold are conventionally considered below the protective threshold [5,6,7].

Several studies have reported a progressive decline in anti-HBs titers over time, particularly among individuals vaccinated during childhood, resulting in a substantial proportion of medical students and medical residents presenting anti-HBs levels below the conventional protective threshold at the start of their professional healthcare careers [5,8,9,10,11,12,13,14,15,16,17,18,19]. However, waning antibody levels are a well-recognized phenomenon and do not necessarily indicate loss of protection in individuals who achieved an adequate immune response after primary vaccination. The clinical interpretation of low or undetectable anti-HBs titers remains controversial. Numerous studies have shown that the absence of detectable antibodies does not necessarily indicate a loss of immunity, as immunological memory—particularly the cellular immune response—can persist for decades after vaccination [20,21,22,23,24,25]. Moreover, most individuals with negative baseline serology exhibit a robust memory response following administration of a booster dose, rapidly achieving protective anti-HBs levels [10,11,12,13,14,15,17,18]. Moreover, several studies have shown that protection against chronic hepatitis B continues even though antibody titers declines [26,27]. In this context, the main clinical concern is the identification of individuals who fail to mount an adequate response to vaccination, as these primary non-responders may remain susceptible to infection. Moreover, individuals who do not respond to booster doses are likely to represent this subgroup, highlighting the importance of targeted post-vaccination assessment in healthcare workers.

These findings challenge the use of serology as the sole marker of protection and reignite the debate regarding the necessity and optimal timing of booster doses in healthcare professionals. Current international recommendations regarding hepatitis B booster vaccination in immunocompetent individuals remain heterogeneous. While some health authorities consider that booster doses are unnecessary in individuals who have completed the primary vaccination schedule and developed an adequate initial immune response, others recommend serological screening and targeted booster administration for high-risk occupational groups. This lack of consensus reflects the complexity of interpreting serological markers of immunity and highlights the need for real-world data describing antibody persistence and booster responses in specific populations, such as healthcare trainees.

Despite the relevance of this issue, data from the Spanish context on baseline serological status against HBV and the response to booster vaccination in newly recruited medical resident are limited. Most available studies have been conducted in other countries and healthcare settings [9,10,11,12,13,14,15]. Therefore, descriptive data on baseline immunity and post-booster responses in this population are of interest. The objective of this study was to analyze the baseline serological status against HBV in medical residents at a Spanish tertiary hospital and to evaluate the serological response to a booster dose in those with anti-HBs < 10 mIU/mL, within the context of the ongoing debate on the validity of serology as a marker of vaccine-induced protection.

## 2. Materials and Methods

### 2.1. Study Design and Setting

Retrospective longitudinal observational study based on routinely collected occupational health records. This approach allowed the inclusion of consecutive cohorts of medical residents without interfering with clinical practice or institutional vaccination protocols.

The study followed the STROBE guidelines for reporting observational studies [28]. Recruitment and data collection corresponded to routine baseline serological screening performed at the Preventive Medicine and Public Health Service of San Cecilio University Hospital (HUSC), Granada, Spain, between January 2021 and December 2024, at the start of residency training.

All serological determinations and booster vaccinations were performed as part of the standard occupational health protocol. The present research consisted of a secondary analysis of anonymized registry data; therefore, no additional procedures or interventions were performed for research purposes. Residents without available serology results were excluded.

### 2.2. Variables and Data Collection

The primary outcome of the study was the antibody titers against the HBV surface antigen (anti-HBs), determined by serology. Testing for anti-HBs was performed in the microbiology laboratory of our hospital center using standardized procedures according to institutional protocols. Anti-HBs levels ≥ 10 mIU/mL were considered to meet the conventional protective threshold, while values < 10 mIU/mL were considered below this threshold [5,6,7]. Therefore, this variable was used qualitatively (dichotomous). The independent variables collected included sex, age, year of residency and specialty. In addition, measles serology was recorded as part of the routine vaccination screening performed for medical residents upon entry to the hospital. This variable was collected to allow comparisons with HBV serology. Measles IgG was determined using a chemiluminescent immunoassay (CLIA) according to the hospital laboratory protocol. Results were reported as an index value (signal-to-cutoff ratio), with values ≥ 1 considered positive and <1 considered negative, following the manufacturer’s instructions. No equivocal or borderline category was recorded in the institutional registry. Measles vaccination history (including number of doses and timing of vaccination) was not available in the institutional registry. For descriptive purposes, age was used quantitatively and also categorized into two groups (<25 and ≥25 years). Age was dichotomized using a cutoff of 25 years based on professional and cohort-related considerations. In our setting, individuals younger than 25 years typically correspond to first-year medical residents undergoing routine occupational health screening at the beginning of their clinical activity, in accordance with standard clinical practice. In contrast, participants older than 25 years represent a more heterogeneous group, including residents with previous professional experience, delayed entry into residency, or prior training pathways. This distinction was considered relevant, as differences in occupational exposure history and time since initial vaccination may influence baseline anti-HBs levels and immune response patterns. Therefore, the age cutoff was used as a pragmatic proxy to differentiate between more homogeneous newly incorporated healthcare workers and those with more variable professional trajectories.

Finally, specialties were classified as medical, surgical, medical–surgical, or laboratory. Data were recorded anonymously using a numerical code assigned to each participant to ensure confidentiality.

### 2.3. Booster Vaccination and Follow-Up

Medical residents with anti-HBs < 10 mIU/mL at baseline were administered a booster dose of the standard HBV vaccine (Engerix-B 20 μg, a recombinant inactivated vaccine), following the standard protocol of the Preventive Medicine Service. A second serological test was then performed at two months to assess the antibody response after the booster dose.

### 2.4. Statistical Analysis

A descriptive analysis of the sample was initially performed. Qualitative variables were expressed as absolute frequencies (*n*) and percentages (%), while quantitative variables were described using mean and standard deviation. Descriptive percentage of medical residents with anti-HBs < 10 mIU/mL at baseline was reported.

Subsequently, a bivariate analysis was conducted. The characteristics of the sample were stratified by baseline anti-HBs levels (≥10 mIU/mL vs. <10 mIU/mL). Chi-square tests were performed for qualitative variables and Student’s *t*-tests for quantitative variables. Conditions of application of all tests were assessed.

Crude and adjusted odds ratios (ORs) were also calculated from multivariable logistic regression models, with their corresponding 95% confidence intervals (95% CIs). Covariates used in adjustments were sex, age (quantitative) and medical specialty. Conditions of application and model validation were assessed.

Finally, a characterization of medical residents that received a booster dose was conducted. Bivariate and multivariable analysis were repeated using “serologic response to booster dose” as dependent variable.

All statistical analyses were performed using Stata (StataCorp^®^, College Station, TX, USA), version 15.0.

### 2.5. Ethical Considerations

This study was approved by the Provincial Research Ethics Committee of Granada, Spain (SICEIA-2025-001330; approval date 30 June 2025). The approval covered the retrospective analysis of anonymized institutional records collected by the Preventive Medicine and Public Health Service between 2021 and 2024. Data were fully anonymized prior to analysis, and all identifiable variables were removed. The study complied with ethical guidelines and was conducted in accordance with the principles of the Declaration of Helsinki.

## 3. Results

### 3.1. Selection and Characteristics of the Sample

**Figure 1:**
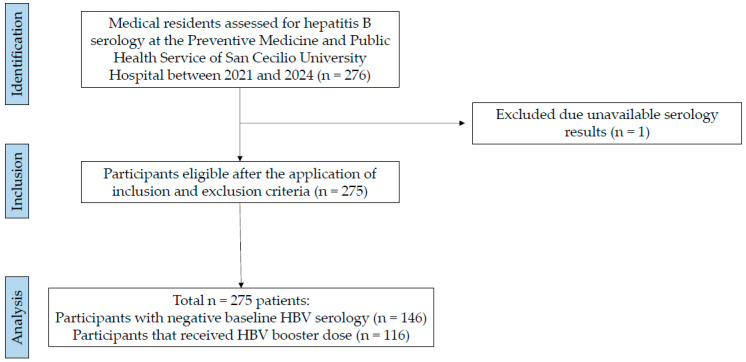
Flowchart of the study selection process.

During the study period, 276 medical residents were registered and requested to undergo HBV serology testing at the Preventive Medicine and Public Health Service of San Cecilio University Hospital. One participant was excluded due to unavailable serological results, resulting in a final sample of 275 (Figure 1).

Table 1 shows the characteristics of the sample stratified by baseline anti-HBs serology. The mean age of the medical residents was 25.4 years (standard deviation = 2.3), and 176 (64.0%) were female. Medical specialties predominated (51.6%), followed by medical–surgical specialties (22.1%), laboratory specialties (17.5%), and surgical specialties (8.7%). The distribution by year of residency was relatively homogeneous: 27.6% were first-year residents (R1), 27.6% second-year residents (R2), 22.6% third-year residents (R3), and 22.2% fourth-year residents (R4). Measles serology was performed in 98.2% of participants, of whom 54.6% tested negative.

### 3.2. Baseline HBV Serological Status

Baseline serological testing showed that 146 medical residents (53.1%) had anti-HBs levels < 10 mIU/mL. Sex and measles serostatus were not associated with baseline anti-HBs levels. Younger residents showed lower frequency of anti-HBs protection. Medical specialties showed heterogeneous serological protection.

Table 2 shows the odds ratios for having baseline anti-HBs levels < 10 mIU/mL after adjustment. The model showed that no baseline characteristic was significantly associated with anti-HBs levels < 10 mIU/mL, except for younger age (OR = 0.75; 95% CI: 0.64–0.88). Results of measles serology were not associated with results of anti-HBs serology in either bivariate or in multivariate analyses.

### 3.3. Serological Response After Booster Vaccination

Among the 146 medical residents with anti-HBs < 10 mIU/mL at baseline, 116 (79.5%) received a booster dose of the HBV vaccine. After booster administration, 110 medical residents (94.8%) achieved anti-HBs ≥ 10 mIU/mL, while 6 (5.2%) with anti-HBs < 10 mIU/mL at baseline remained below the protective threshold. The serological response following booster vaccination and the characteristics associated with seroconversion are shown in Table 3.

## 4. Discussion

In this longitudinal observational study, more than half of the medical residents were found to have anti-HBs < 10 mIU/mL at baseline despite having been vaccinated against HBV according to the national immunization program. However, following administration of a booster dose, the vast majority of residents with anti-HBs levels < 10 mIU/mL at baseline achieved anti-HBs ≥ 10 mIU/mL after booster administration, supporting the persistence of immunological memory.

The use of routinely collected data reflects real-world occupational health procedures and provides a pragmatic assessment of HBV immunity status in newly incorporated healthcare professionals.

The high percentage of baseline anti-HBs levels < 10 mIU/mL observed in this cohort is consistent with previous reports in similar populations of medical students and young healthcare professionals vaccinated in early life according to national immunization schedules [8,9,10,11,12,13,14,15,16,17,18]. Several studies have documented a progressive decline in anti-HBs antibody levels over time, particularly when primary vaccination is administered at an early age [5,29,30]. This phenomenon has raised concerns in groups at high occupational risk, such as medical residents, who may begin their healthcare careers with low or undetectable antibody titers. In the Spanish context, individuals born from the early 1990s received only the infant HBV vaccination schedule and did not receive booster doses during adolescence. This may partially explain the high prevalence of low antibody titers in this population; however, individual vaccination histories were not available, and cohort-related differences should therefore be interpreted with caution.

Similar findings have been reported in multiple international studies evaluating HBV immunity among medical students and young healthcare workers. Research conducted in Europe [31], Asia [32], and North America [15] has consistently shown that a substantial proportion of individuals vaccinated during infancy present anti-HBs levels below the conventional protective threshold by early adulthood. In several of these studies, the proportion of individuals with anti-HBs < 10 mIU/mL ranged from approximately 30% to over 60%, depending on the population studied and the time elapsed since primary vaccination. These observations suggest that declining circulating antibody levels represent a common immunological pattern rather than a phenomenon specific to a single healthcare system or country.

Nevertheless, the clinical relevance of these low titers remains a matter of debate. The high response rate observed after administration of a booster dose in our study is consistent with previous reports and supports the hypothesis that a decline in circulating antibodies does not necessarily indicate a loss of protection against HBV [10,11,12,13,14,15,17,18]. The rapid increase in anti-HBs titers following booster vaccination is indicative of the persistence of immunological memory, likely mediated by specific humoral and cellular mechanisms against the surface antigen.

In this context, several studies have questioned the use of anti-HBs serology as the sole marker of vaccine-induced protection. It has been reported that circulating antibody levels do not always correlate with their neutralizing capacity against HBV [20]. Similarly, studies assessing the cellular immune response have demonstrated the persistence of long-term immunological memory, even in individuals with anti-HBs titers < 10 mIU/mL [21,22,23,24,25,33,34]. These findings reinforce the notion that serology reflects only part of the immune status and may underestimate the actual protection against infection. From an immunological perspective, the persistence of protection after hepatitis B vaccination is largely attributed to the maintenance of immunological memory rather than to sustained high circulating antibody levels. Memory B cells and antigen-specific T lymphocytes can rapidly expand following antigen re-exposure, leading to a rapid increase in anti-HBs production after booster administration. This anamnestic response has been widely documented and is considered a key indicator of preserved vaccine-induced immunity. Consequently, the absence of detectable antibodies should not automatically be interpreted as a complete loss of protection, particularly in individuals with documented prior vaccination.

In our study, an association was observed between younger ages and a lower frequency of positive baseline serology. This finding could be due to chance or may be explained by other mechanisms, such as variations in vaccine effectiveness across different cohorts, potential differences in immune response between generations, or unmeasured confounding factors. In any case, as this is a descriptive study aimed at characterizing the problem, future mechanistic studies will be needed to explore the causes of this apparent difference between individuals of similar ages. The differences observed between medical specialties are, to the best of our knowledge, stochastic or attributable to residual confounding. It is important to note that no differences were observed in the characteristics of the residents with respect to their response to the booster dose, as the vast majority responded successfully. Furthermore, no relationship was observed between baseline HBV serology and measles serology, suggesting that a negative HBV baseline result was unlikely to be attributable to a generalized immune impairment, poor adherence to the national vaccination schedule during early life, or impaired serological response, but rather reflects independent associations that require further investigation.

The relatively high proportion of measles seronegativity observed in our cohort warrants cautious interpretation. Measles IgG was assessed using a qualitative CLIA index-based result, without quantitative antibody titers, vaccination history, or confirmatory neutralization assays available for further analysis. Importantly, immunity gaps in young adults have been described in European settings. Recent data from Austria reported seronegativity rates between 13% and 20% among individuals born after 1990 [35], and a study conducted in southern Italy found serosusceptibility of 39.8% among young adults aged 18–24 years [36]. Therefore, although the proportion observed in our cohort appears high, similar trends have been documented in comparable age groups, suggesting that declining detectable circulating IgG levels rather than absence of immunological memory may partially explain this finding.

These findings have important implications for occupational health policies in healthcare institutions. As cohorts vaccinated during infancy continue to enter the healthcare workforce, hospitals increasingly encounter professionals with low or undetectable anti-HBs titers at baseline screening. Establishing evidence-based strategies to manage this situation is therefore essential. Approaches based on systematic baseline serology followed by targeted booster vaccination may represent a pragmatic solution that balances occupational protection with efficient use of healthcare resources. From a practical perspective, the results of this study support the usefulness of performing baseline serological screening at the start of medical residency, with the aim of identifying those professionals with anti-HBs levels anti-HBs < 10 mIU/mL and offering them a booster dose. This strategy would strengthen humoral protection in a group with high occupational risk and is consistent with HBV prevention recommendations for healthcare personnel, as well as aligning with the hepatitis B elimination targets proposed by the World Health Organization for 2030 [37]. Ensuring adequate protection against HBV among healthcare professionals also contributes indirectly to broader public health goals. Healthcare workers play a critical role in preventing nosocomial transmission and maintaining patient safety. Maintaining high levels of immunity in this group supports global hepatitis elimination strategies and reinforces infection prevention policies within healthcare systems.

This study has several limitations that should be considered. A major limitation is the absence of detailed vaccination history or access to vaccination certificates during infancy. Information regarding the number of hepatitis B vaccine doses received, the time elapsed since the last vaccine dose, the specific vaccination schedule (infant versus adolescent), and whether vaccination status was documented or self-reported was not consistently available in the institutional records. As Spain transitioned from targeted and adolescent vaccination programs to universal infant immunization during the 1990s, cohort effects may substantially influence long-term antibody persistence. The same situation occurs with respect to previous measles vaccination. Consequently, it cannot be definitively determined whether anti-HBs < 10 mIU/mL in this cohort reflect declining circulating antibody concentrations after complete primary vaccination, incomplete vaccination schedules, or differences related to vaccination strategies implemented across birth cohorts. This limitation should be carefully considered when interpreting the findings.

Additionally, this is a single-center study, which may limit the generalizability of the results. Information on other potentially relevant variables, such as the exact time elapsed since primary vaccination or the presence of comorbidities that could influence immune response, was not available in our registries. Furthermore, approximately 20% of medical residents with anti-HBs levels < 10 mIU/mL did not attend the appointment to receive the booster vaccination, highlighting the need to improve adherence to institutional vaccination protocols.

Another limitation relates to the use of anti-HBs serology as the sole immunological marker. Cellular immune responses and memory B-cell activity were not assessed in this study. As these components play a critical role in long-term vaccine-induced protection, future studies incorporating immunological assays beyond antibody titers would provide a more comprehensive understanding of HBV immunity persistence in healthcare workers.

Future research should include multicenter designs and consider incorporating markers of cellular immune response to more accurately assess long-term protection against HBV. It would also be valuable to investigate whether alternative strategies, such as targeted administration of booster doses or optimization of vaccination schedules, could improve long-term protection in healthcare professionals.

## 5. Conclusions

This study found that a high proportion of medical residents had anti-HBs levels below the conventional protective threshold (<10 mIU/mL) at the start of their healthcare careers, despite having been vaccinated against HBV according to the national immunization program. However, the vast majority of those with anti-HBs levels < 10 mIU/mL at baseline developed an adequate antibody response following a single booster dose, supporting the persistence of immunological memory.

These findings support the utility of baseline serological screening combined with selective booster vaccination as an effective strategy to enhance humoral protection against HBV in newly recruited healthcare professionals. They also highlight that anti-HBs serology alone may not fully reflect the actual immune status, underscoring the need for further research into alternative immunological markers to optimize hepatitis B prevention and control strategies in healthcare settings.

## Figures and Tables

**Table 1:** Baseline characteristics of the study sample and baseline stratified by anti-HBs serological status (*n* = 275).

Variable	Total, *n* (%)/ Mean (sd)	Anti-HBs ≥ 10 mIU/mL, *n* (%)	Anti-HBs < 10 mIU/mL, *n* (%)	*p*-Value
**Total**	275 (100.0%)	129 (46.9%)	146 (53.1%)	-
**Sex**				0.386
Female	176 (64.0%)	86 (48.9%)	90 (51.1%)	
Male	99 (36.0%)	43 (43.4%)	56 (56.6%)	
**Age (quantitative)**	25.4 (2.3)	26.2 (3.1)	25.0 (1.6)	0.002
**Age (groups)**				0.367
<25 years	97 (35.3%)	42 (43.3%)	55 (56.7%)	
≥25 years	178 (64.7%)	87 (48.9%)	91 (51.1%)	
**Specialty**				0.022
Medical	142 (51.6%)	72 (50.7%)	70 (49.3%)	
Medical-surgical	61 (22.1%)	33 (54.1%)	28 (45.9%)	
Laboratory	48 (17.5%)	19 (39.6%)	29 (60.4%)	
Surgical	24 (8.7%)	5 (20.8%)	19 (79.2%)	
**Residency year**				0.002
First (R1)	76 (27.6%)	29 (38.2%)	47 (61.8%)	
Second (R2)	76 (27.6%)	27 (35.5%)	49 (64.5%)	
Third (R3)	62 (22.6%)	40 (64.5%)	22 (35.5%)	
Fourth (R4)	61 (22.2%)	33 (54.1%)	28 (45.9%)	
**Measles serology**				0.827
Positive	152 (56.1%)	71 (46.7%)	81 (53.3%)	
Negative	119 (43.9%)	54 (45.4%)	65 (54.6%)	

**Table 2:** Odds ratio of baseline anti-HBs < 10 mIU/mL in medical residents. All variables of the table were used as covariates for a multivariable logistic regression model.

Variable	Crude (Unadjusted) OR (95 CI%)	Adjusted OR (95 CI%)
**Sex**		
Female	0.85 (0.54–1.32)	0.66 (0.37–1.52)
Male	Reference	Reference
**Age (quantitative)**	0.81 (0.73–0.91)	0.75 (0.64–0.88)
**Age (groups)**		
<25 years	Reference	Reference
≥25 years	0.79 (0.49–1.31)	1.66 (0.85–3.26)
**Specialty**		
Medical	Reference	Reference
Surgical	3.91 (1.38–11.04)	3.75 (1.27–11.06)
Medical-surgical	0.87 (0.48–1.59)	0.72 (0.38–1.38)
Laboratory	1.57 (0.81–3.05)	1.61 (0.76–3.41)
**Positive measles serology**	0.95 (0.59–1.53)	0.87 (0.51–1.49)
First (R1)	76 (27.6%)	29 (38.2%)
Second (R2)	76 (27.6%)	27 (35.5%)

**Table 3:** Characteristics of the sample stratified by serological response after HBV booster vaccination among medical residents with anti-HBs < 10 mIU/mL at baseline (*n* = 116).

Variable	Total, *n* (%)/ Mean (sd)	Anti-HBs ≥ 10 mIU/mL, *n* (%)	Anti-HBs < 10 mIU/mL, *n* (%)	*p*-Value
**Total**	116 (100.0%)	110 (94.8%)	6 (5.2%)	-
**Sex**				0.386
Female	72 (62.1)	69 (95.8%)	3 (4.2%)	
Male	44 (37.9)	41 (93.2%)	3 (6.8%)	
**Age (quantitative)**	25.0 (1.7)	24.7 (0.8)	25.0 (1.7)	0.583
**Age (groups)**				0.672
<25 years	44 (37.9)	41 (93.2%)	3 (6.8%)	
≥25 years	72 (62.1)	69 (95.8%)	3 (4.2%)	
**Specialty**				0.375
Medical	52 (44.8%)	47 (93.4%)	5 (9.6%)	
Surgical	14 (12.1%)	14 (100.0%)	0 (0.0%)	
Medical-surgical	24 (20.7%)	24 (100.0%)	0 (0.0%)	
Laboratory	26 (22.4%)	25 (96.2%)	1 (3.9%)	
**Residency year**				0.559
First (R1)	33 (28.5%)	30 (90.9%)	3 (9.1%)	
Second (R2)	40 (34.5%)	38 (95.0%)	2 (5.0%)	
Third (R3)	20 (17.2%)	19 (95.0%)	1 (5.0%)	
Fourth (R4)	33 (19.8%)	23 (100.0%)	0 (0.0%)	
**Measles serology**				0.693
Positive	65 (56.0%)	61 (93.9%)	4 (6.2%)	
Negative	51 (43.9%)	49 (96.1%)	2 (3.9%)	

## Data Availability

Data will be available upon reasonable request to the corresponding author.

## Abbreviations

The following abbreviations are used in this manuscript:

HBs	Hepatitis B virus surface antigen
HBV	Hepatitis B virus
HUSC	Hospital Universitario San Cecilio
STROBE	Strengthening the Reporting of Observational Studies in Epidemiology
OR	Odds Ratio
R1	Medical resident during the first year of medical training
R2	Medical resident during the second year of medical training
R3	Medical resident during the third year of medical training
R4	Medical resident during the fourth year of medical training
